# Perceptual conflict during sensorimotor integration processes - a neurophysiological study in response inhibition

**DOI:** 10.1038/srep26289

**Published:** 2016-05-25

**Authors:** Witold X. Chmielewski, Christian Beste

**Affiliations:** 1 Cognitive Neurophysiology, Department of Child and Adolescent Psychiatry, Faculty of Medicine of the TU Dresden, Germany

## Abstract

A multitude of sensory inputs needs to be processed during sensorimotor integration. A crucial factor for detecting relevant information is its complexity, since information content can be conflicting at a perceptual level. This may be central to executive control processes, such as response inhibition. This EEG study aims to investigate the system neurophysiological mechanisms behind effects of perceptual conflict on response inhibition. We systematically modulated perceptual conflict by integrating a Global-local task with a Go/Nogo paradigm. The results show that conflicting perceptual information, in comparison to non-conflicting perceptual information, impairs response inhibition performance. This effect was evident regardless of whether the relevant information for response inhibition is displayed on the global, or local perceptual level. The neurophysiological data suggests that early perceptual/ attentional processing stages do not underlie these modulations. Rather, processes at the response selection level (P3), play a role in changed response inhibition performance. This conflict-related impairment of inhibitory processes is associated with activation differences in (inferior) parietal areas (BA7 and BA40) and not as commonly found in the medial prefrontal areas. This suggests that various functional neuroanatomical structures may mediate response inhibition and that the functional neuroanatomical structures involved depend on the complexity of sensory integration processes.

During sensorimotor integration, the ability to inhibit responses is central to behavioral control and is assumed to represent a core executive control process^1^. To successfully deploy inhibitory control processes, perceptual and attentional processes are essential to inform sensorimotor integration processes during response inhibition^2^. This has also been suggested by latent variable analyses^3^ indicating that response inhibition performance correlates with resistance to distractor interference. However, in a complex environment the relevant information used for sensorimotor integration may be more or less detectable. It is well-known that a crucial factor for detecting the relevant information is, whether the “bigger picture”, i.e. the global (holistic) level contains the relevant information, or whether the details, i.e. the local level has to be attended^4^,^5^. This is usually examined in Global-local tasks where people react based on the information displayed via ambiguous stimuli: e.g. a capital letter “H” that is built up by small “S” letters. The participant’s task is to respond according to the global (H letter), or the local dimension (S letters)^6^,^7^. With this approach, and depending on the employed experiments, instructions and stimulus material, an advantage for either the global^5^,^6^,^7^, or the local processing dimension has been observed^8^,^9^. However, usually it is the global processing dimension that seems to dominate over local processing of stimulus features^10^.

Yet, currently it is not clear what neuronal mechanisms may relate to possible effects of perceptual conflict on subsequent response inhibition processes and what processing dimension exerts stronger effects on subsequent response inhibition processes. Stock *et al.*^11^ for example showed that the behavioral outcome of response of response inhibition greatly depends on the amount of resources allocated to early stages of stimulus-response activation during responding, suggesting to focus more on early processing steps. Likewise, Verbruggen *et al.*^12^ showed that perceptual interference effects, as seen in Global-local tasks, affect performance in a stop-signal task in a way that perceptual interference prolongs stop-signal reaction times and thus compromises inhibitory control processes. Verbruggen *et al.*^12^ argue that this prolongation demonstrates that response inhibition interacts with interference control at early processing stages. However, Shedden and Reid^10^ using a cued response selection Global-local task with variable mappings between the stimulus and the response, showed that the global and the local stimulus dimension can have similar effects on response speed. The authors argue that for response control processes requiring a more deliberate processing of stimuli, interferences takes places after perceptual processing stages^10^. As response inhibition also refers to response control, it is conceivable that inference is evident at later processing stages. Yet, there is currently no clear neurophysiological evidence, or information about functional neuroanatomical structures related to the mechanisms of perceptual interference effects on response inhibition processes. Using EEG-methods in combination with source localization techniques, it is possible to examine at what processing level perceptual conflicts interfere with response inhibition processes and what functional neuroanatomical structures are involved. In the current study, this is done using a combined “Global-local-Go/Nogo task”.

On a behavioral level we expect the false alarm rate (i.e. the rate of not withheld responses on Nogo-trials) to be smallest when information carried via the global and the local stimulus dimension match. False alarm rates may increase when there is a conflict between the global and local stimulus dimension. If the effect of global and local stimulus dimensions occurs at the initial perceptual evaluation of the stimulus (i.e. at the response selection level), event-related potentials (ERPs) reflecting perceptual and attentional gating processes (i.e. P1 and N1) will be affected. Yet, it has been shown that perceptual conflicts modulate medial frontal brain areas^13^,^14^,^15^,^16^, also known to mediate response inhibition processes^17^,^18^,^19^,^20^,^21^. It is therefore also likely that the latter, and not modulations at early perceptual and attentional processing stages, relate to effects of perceptual interference on response inhibition processes. However, as especially parietal areas have been suggested to bridge perception and action^22^ and may update internal representations using sensory information to initiate appropriate actions^23^, it cannot be excluded that parietal areas are related to neurophysiological processes of response inhibition as well. On a neurophysiological level, response inhibition subprocesses are reflected by the Nogo-N2 and Nogo-P3. These neurophysiological correlates have been suggested to reflect conflict and/or pre-motor inhibition processes (Nogo-N2)^24^,^25^,^26^,^27^,^28^,^29^ and, in case of the Nogo-P3, the inhibition process per se and/or evaluation processes of the successful outcome of an inhibition^2^,^30^,^31^,^32^,^33^,^34^,^35^,^36^. Regarding the behavioral effects it is, however, likely that the Nogo-N2 and Nogo-P3 are decreased in amplitude, because compromised inhibition performance has repeatedly been shown to be associated with smaller Nogo-N2 and Nogo-P3 amplitudes. Moreover, Shedden and Reid^10^ suggested that the normal global precedence effect (i.e. the global dimension has a stronger impact than the local dimension) is not evident at the response selection level. If modulatory effects emerge at the response selection and not the perceptual level, it is likely that the global and local dimension of a stimulus will have similar strength to modulate response inhibition processes.

## Results

### Behavioral data

Concerning Go trials, no significant differences could be detected in Go_redundant_ (98.6 ± 0.4) and Go (98.3 ± 0.5) hit rates (t(33) = 1.13; p = 0.266). However, RTs on Go_redundant_ (577 ± 16) trials were significantly shorter than on Go trials (587 ± 16) (t(33) = −2.87, p = 0.007).

The false alarms (FA) rate is the most important behavioral parameter in Go/Nogo tasks. For the FA rates the ANOVA revealed a main effect of condition (F(2, 66) = 115.18; p < 0.001; η^2^ = 0.777), showing FA rates to be lower in the Nogo_redundant_ (5.3 ± 0.8), as compared to the Nogo_global_ (25.3 ± 0.1.8) and Nogo_local_ (27.2 ± 1.5) condition. Post-hoc paired t-tests revealed Nogo_redundant_ FA rates to be significantly decreased in comparison to the other two conditions (all t ≥ 12.35; p < 0.001). However, no significant differences were found between the Nogo_global_ and Nogo_local_ (t(33) = −1.05; p = 0.304). A similar pattern was found in the ANOVA for the RTs on responses on Nogo trials (F(2, 66) = 29.02; p < 0.001; η^2^ = 0.509). Again, post-hoc paired t-tests revealed RTs to be shorter in the Nogo_redundant_ (468 ± 13) condition in comparison to the other two conditions (all t ≥ 5.60; p < 0.001), while between Nogo_global_ (569 ± 17) and Nogo_redundant_ (580 ± 18) trials no significant differences were found (t(33) = −0.851; p = 0.401). However, the pattern of FA RTs might be directly related to the FA rates, meaning outliers should have a bigger impact on the Nogo_redundant_ condition and thus bias the RTs.

### Neurophysiological data

#### Early processing stage

**Figure 1:**
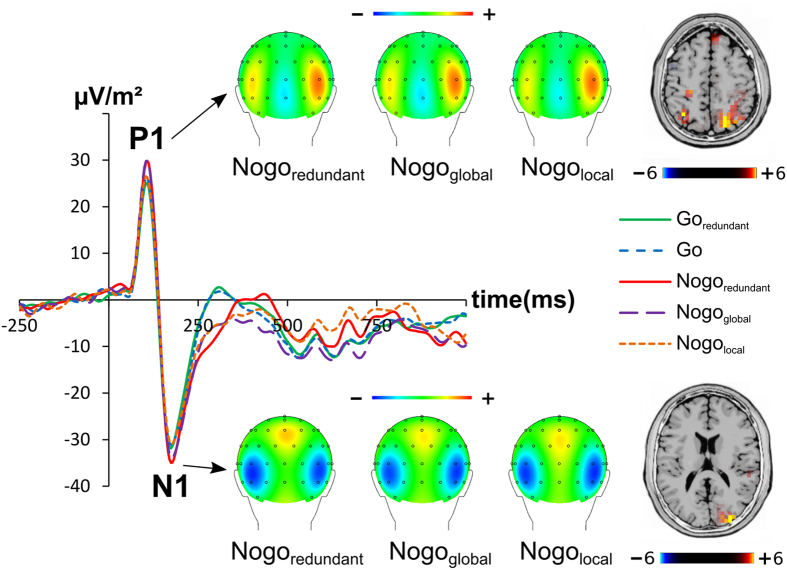
P1 and N1 amplitudes averaged across electrodes P7 and P8. The y-axis denotes μV/m^2^ and the x-axis denotes the time in ms. Time point zero denotes the time point of stimulus presentation. The different lines show Go_redundant_ (green), Go (blue), Nogo_redundant_ (red), Nogo_global_ (purple) and Nogo_local_ (orange). The CSD scalp topography plots show the distribution of the scalp electrical potential for the P1 (upper row) and N1 (lower row) on NoGo trials. Warm colors indicate positive and cold colors negative deflections. The corresponding P1 and N1 sLORETA plots are shown besides the respective topography plots and denote the activation differences between the Nogo_redundant_ and Nogo_local_ condition. The color scale denotes the critical t-values.

The P1 and N1 ERPs are shown in Fig. 1.

Concerning the P1 amplitude the ANOVA revealed a main effect of condition (F(4, 264) = 16.35; p < 0.001; η^2^ = 0.198), showing the P1 amplitude to increase from the Go_redundant_ (29.4 ± 2.6 μV/m^2^) to the Go (30.6 ± 2.4 μV/m^2^),to the Nogo_local_ (31.6 ± 2.8 μV/m^2^), to the Nogo_redundant_ (35.0 ± 2.7 μV/m^2^), to the Nogo_global_ (35.2 ± 2.6 μV/m^2^) condition. This was confirmed by post-hoc paired t-tests showing that the Go trial amplitude was significantly smaller (i.e. less positive) than the Nogo_redundant_ and the Nogo_global_ amplitude (all t ≥ 4.72; p < 0.001), while the Go_redundant_ amplitude was significantly smaller (i.e. less positive) than all three Nogo trial amplitudes (all t ≥ 2.12; p < 0.042). Additionally, the Nogo_local_ amplitude was significantly smaller (i.e. less positive) than the other two Nogo amplitudes (all t ≥ 2.96; p < 0.006). Using sLORETA we contrasted the Nogo_global_ condition against the Nogo_local_ condition. The analysis showed that areas in the precuneus and superior parietal lobe (BA7) were more activated in the Nogo_global_ condition than in the Nogo_local_ condition (refer Fig. 1). The sLORETA analysis was restricted to these conditions to account for how much perceptual processes and functional neuroanatomical structures are modulated. Moreover, a main effect of electrode (F(1, 66) = 235.17; p < 0.001; η^2^ = 0.781) was revealed, showing the P1 amplitudes to be smaller (i.e. less positive) at electrode P8 than at electrode P7. The interaction “condition x electrode” showed no significant differences (F(4, 264) = 0.82; p < 0.513; η^2^ = 0.012).

For the N1 amplitudes the ANOVA revealed a main effect of condition (F(4, 264) = 6.44; p < 0.001; η^2^ = 0.89), showing the N1 amplitudes to become more negative from the Go_redundant_ (−40.5 ± 3.9 μV/m^2^) to the Nogo_local_ (−41.0 ± 3.8 μV/m^2^), to the Go (−41.1 ± 4.0 μV/m^2^), to the Nogo_global_ (−43.9 ± 4.4 μV/m^2^), to the Nogo_redundant_ (−45.7 ± 4.3 μV/m^2^) condition. This was confirmed by post-hoc paired t-tests revealing that amplitudes in the two Go conditions were smaller (i.e. less negative) than the Nogo_global_ and the Nogo_redundant_ amplitudes (all t ≥ 2.21; p < 0.034). Furthermore, the Nogo_local_ amplitude was significantly smaller (i.e. less negative) than the Nogo_redundant_ amplitude (t(33) = 3.12, p = 0.004). Using sLORETA we contrasted the Nogo_redundant_ condition against the Nogo_local_ condition. The analysis showed that an area in the middle occipital gyrus (BA19) was more activated in the Nogo_redundant_ condition than in the Nogo_local_ condition (refer Fig. 1). Moreover, a main effect of electrode (F(1, 66) = 201.00; p < 0.001; η^2^ = 0.753) was observed, showing the N1 amplitudes to be smaller (i.e. less negative) at P7 than at P8. The interaction “condition x electrode” showed no significant differences (F(4, 264) = 0.33; p = 311; η^2^ = 0.016).

### Response selection stage

**Figure 2:**
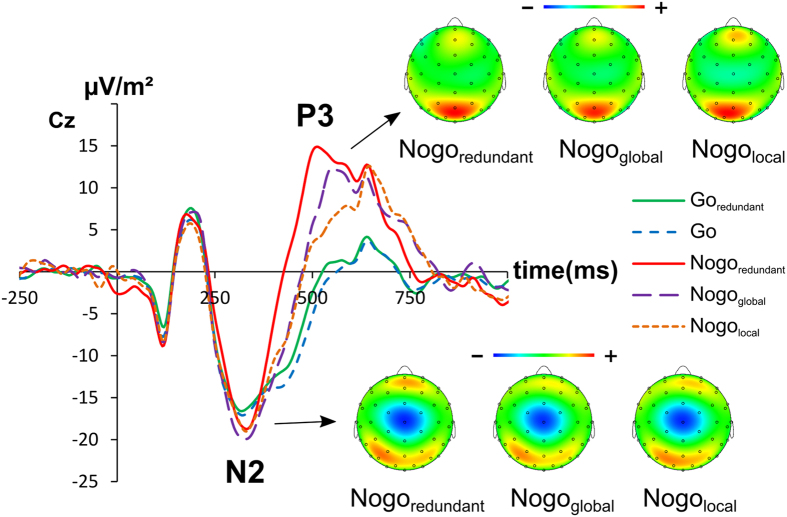
N2 amplitude at electrode Cz. The y-axis denotes μV/m^2^ and the x-axis denotes the time in ms. Time point zero denotes the time point of stimulus presentation. The different lines show Go_redundant_ (green), Go (blue), Nogo_redundant_ (red), Nogo_global_ (purple) and Nogo_local_ (orange). The topography plots show the distribution of the scalp electrical potential for the N2. Warm colors indicate positive and cold colors negative deflections. Additionally the topography plots for the P3 are displayed.

**Figure 3:**
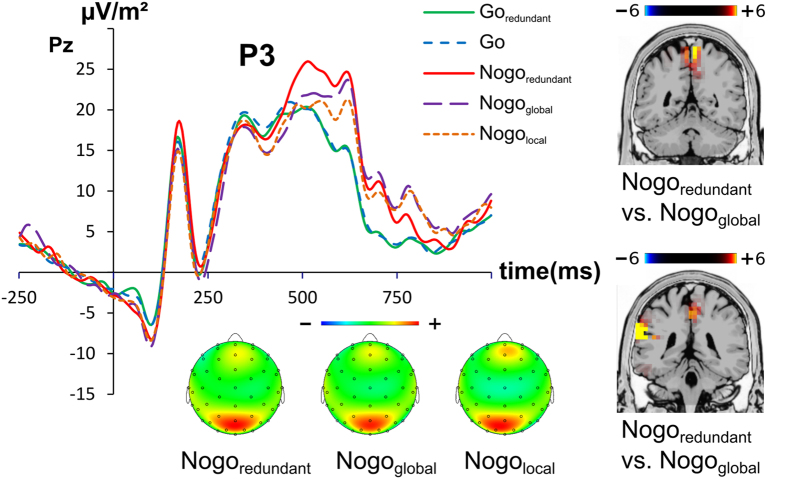
P3 amplitude at electrode Pz. The y-axis denotes μV/m^2^ and the x-axis denotes the time in ms. Time point zero denotes the time point of stimulus presentation. The different lines show Go_redundant_ (green), Go (blue), Nogo_redundant_ (red), Nogo_global_ (purple) and Nogo_local_ (orange). The topography plots show the distribution of the scalp electrical potential P3 (note that the scaling is slightly different between the conditions). Warm colors indicate positive and cold colors negative deflections. The P3 sLORETA plots are shown at the right side of the figure and denote the activation differences between the Nogo_redundant_ vs. Nogo_global_ (upper plot) and Nogo_redundant_ vs. Nogo_local_ (lower plot) condition. The color scale denotes the critical t-values.

The N2 and P3 ERPs are shown in Figs 2 and 3.

Concerning the N2 amplitude at Cz the ANOVA revealed a main effect of condition (F(4, 132) = 6.51; p < 0.001; η^2^ = 0.165), showing the N2 amplitudes to become more negative from the Go_redundant_ (−20.9 ± 2.1), to the Go (−21.7 ± 2.1), to the Nogo_local_ (−23.5 ± 2.6), to the Nogo_redundant_ (−24.4 ± 2.5), to the Nogo_global_ (−26.1 ± 2.2) condition. Post-hoc paired t-tests revealed that the N2 was smaller (i.e. less negative) in the two Go conditions in comparison to all Nogo conditions (all t ≥ 1.82, p ≤ 0.039). Furthermore, Nogo_global_ amplitude was bigger (i.e. more negative) than the Nogo_local_ amplitude (t(33) = −2.03, p = 0.025), which resembles the effects seen at the perceptual level (P1 and N1). All other post-hoc paired t-tests were not significantly different from each other (all t ≤ 1.52, p ≥ 0.070).

As indicated by the scalp topography plots the (Nogo-)P3 was not maximal on fronto-central electrode sites, but on parietal electrode sites Therefore, the P3 was analyzed at electrode Pz. For the P3 amplitude, the ANOVA revealed a main effect of condition (F(4, 132) = 9.22; p < 0.001; η^2^ = 0.218), showing the P3 amplitude to increase from the Go_redundant_ (18.5 ± 2.4), to the Go (18.9 ± 2.5), to the Nogo_local_ (20.1 ± 3.1), to the Nogo_global_ (21.0 ± 3.0), to the Nogo_redundant_ (24.1 ± 2.9) condition. Post-hoc paired t-tests revealed the two Go condition’s amplitudes to be smaller (i.e. less negative) than the Nogo_redundant_ and Nogo_global_ amplitudes (all t ≥ 2.30, p ≤ 0.028). Furthermore the Nogo_redundant_ amplitude was bigger (i.e. more positive) than the Nogo_global_ and Nogo_local_ amplitude (all t ≥ 2.80, p ≤ 0.009). All other post-hoc paired t-tests did not significantly differ from each other (all t ≤ 1.39, p ≥ 0.178). Using sLORETA we contrasted the Nogo_redundant_ against the Nogo_global_ and the Nogo_redundant_ against the Nogo_local_ in two separate contrasts (i.e. Nogo_redundant_ > Nogo_global_ and Nogo_redundant_ > Nogo_local_). For the contrast Nogo_redundant_ > Nogo_global_ the analysis showed activation differences in the precuneus and the postcentral gyrus (BA7). For the contrast Nogo_redundant_ > Nogo_local_ the analysis also showed activation differences in the precuneus and the postcentral gyrus (BA7), but also activation differences in the inferior parietal cortex (temporo-parietal junction, TPJ) (BA40) (refer Fig. 3).

## Discussion

In the current study, we examined the role of perceptual conflict for response inhibition processes with a focus on neurophysiological subprocesses and functional neuroanatomical structures being modulated during this special instance of sensorimotor integration. To do so, we combined elements of a classical Global-local task with a Go/Nogo task. Using Global-local task elements, we manipulated the perceptual dimensions carrying information to respond, or information to withhold from responding. These perceptual dimensions (i.e. the global and the local dimension) could either carry the same information, or could contain conflicting information (i.e., the global dimension carries “Go-information” and the local dimension carries “Nogo-information”, or vice versa). This manipulation creates task conditions where response inhibition has to be carried out under perceptual conflict, or under no perceptual conflict.

The behavioral and neurophysiological results show that response inhibition performance was differentially modulated between perceptual conflict conditions and condition, in which no perceptual conflict was evident. The rate of false alarms was higher in conditions comprising a conflict between the global and the local stimulus dimension, compared the condition where there was no conflict between the stimulus dimensions. This shows that response inhibition performance becomes compromised when there is a conflict between stimulus dimensions, which could be related to an increased decision difficulty in conflicting trials^37^,^38^. This suggests that perceptual factors have a strong influence on response inhibition processes. This has also been suggested in a previous behavioral study^12^. Interestingly, there was no difference between the perceptual conflict conditions. Thus, when these dimensions are conflicting, it does not matter whether the Nogo information is carried via the global stimulus dimension or the local stimulus dimension. There is hence no global precedence effect^5^ when it comes to response inhibition processes, i.e. NoGo trials. Information to inhibit a response is compromised from the conflicting information to execute a response, no matter whether the information to inhibit the response is carried via the global, or the local stimulus dimension. According to Shedden and Reid^10^ this already suggests that the locus of interference is at the response selection and not at early processing stages. This is, in fact, underlined by the neurophysiological data.

Neurophysiological correlates of perceptual and (bottom-up) attentional gating processes (i.e. P1 and N1)^39^ were modulated between the redundant and local condition with the P1 and N1 being stronger in conditions where the Nogo-information is not exclusively carried via the local stimulus dimension. The source localization analysis showed that areas in the precuneus and superior parietal lobe (BA7), as well as the middle occipital gyrus (BA19) are modulated. There areas are known to be involved in perceptual and attentional gating processes^40^,^41^,^42^. However, despite the fact that the global dimension seems to be more salient than the local dimension, this does not differentially affect response inhibition performance, as evidenced by the behavioral data. Differences in the P1 and N1 may thus only reflect perceptual differences between processing of Nogo-information carried via the global and the local dimension. As the global dimension is perceptually more salient, it is associated with larger P1 and N1 amplitudes. Processes downstream perceptual and attentional selection processes, i.e. at the response selection level, seem to be more important for the understanding of modulations in response inhibition processes.

At the response selection level the effects of conflicting global and local stimulus dimension were very specific: no effects were observed for the Nogo-N2, but for the Nogo-P3. This suggests that pre-motor inhibition processes (likely reflected by Nogo-N2)^27^ are not modulated. A different interpretation of the Nogo-N2 is that it reflects the conflict to execute or to inhibit a response^28^,^29^. The current results seem to refute this interpretation, because a clear perceptual conflict is well-known to be evident in the task applied^16^,^43^. However, it is possible that the Nogo-N2 specifically reflects conflict at the response selection, and not at the perceptual level. It rather seems that response inhibition processes^36^, or evaluative processes of response inhibition^31^,^34^,^35^, as reflected by the Nogo-P3, are affected. The Nogo-P3 was highest in the condition with redundant global and local processing dimensions, as compared to the conditions with conflicting global or local processing dimensions. This pattern of modulation is exactly in line with the behavioral data: response inhibition was best in the condition with redundant global and local processing dimensions and comparably worse in the two conditions with conflicting global or local processing dimensions.

However, opposed to what is commonly found for the Nogo-P3^44^,^45^,^46^ the scalp topography was shifted to more posterior (parietal) electrode sites. Accordingly, the source localization analysis using well-validated methods (cf. methods section) revealed that differences between conditions are due to activation differences in the in the precuneus and the postcentral gyrus (BA7) (redundant >global; redundant >local), as well as the left temporo-parietal junction (TPJ, BA40) (redundant >local). This contrasts to most findings in response inhibition where medial prefrontal areas as commonly found^47^. Yet, the critical aspect in the current study is the importance of modulated perceptual processes to provide information for response inhibition processes. This may explain the involvement of parietal brain areas in response inhibition. That is, areas in the posterior and inferior parietal cortex have previously been found to be involved in the Global-local task^48^, possibly because these areas are involved in perceptual integration process^22^. The results therefore suggest that brain areas known to be involved in the resolution of perceptual ambiguities between the global and the local stimulus dimension are also involved in executive control processes (response inhibition) based thereon. This may be possible because parietal areas including BA40 and BA7 have been suggested to bridge perception and action and therefore play central roles in sensorimotor integration processes^22^. Several lines of evidence further suggest that in cases with elevated demands on conflict processing capacities, the superior and inferior parietal cortex (i.e., BA7 and BA40) including the temporo-parietal junction (TPJ)^49^ are involved. This has also be shown during response inhibition^50^. It is also known that BA7 is involved when incoming information is complex, but essential for subsequent behavioral processes^51^,^52^. This fits with notions that the inferior parietal areas may update internal representations using sensory information to initiate appropriate actions^23^. It is therefore possible that due to these processing features parietal structures play a role during response inhibition, when modulated by perceptual conflicts between global and local stimulus dimensions. The P3 amplitude data (i.e. reduced P3 amplitudes in conflicting trials) suggests that the integration of stimulus dimensions in BA7 and BA40 to initiate appropriate actions is insufficient and that the conflict between the global and the local stimulus dimension is not fully resolved, which compromises response inhibition performance as revealed by the behavioral data. Verbruggen *et al.*^12^ suggested that response inhibition interacts with interference control at early processing stages. The neurophysiological data suggests that functional neuroanatomical areas that **are** involved in (early) sensory integration processes are modulated. However, these modulations seem to occur after perceptual and attentional selection processes.

For future studies, it might be reasonable to study psychiatric populations, which are assumed to deviate in the processing of global and local information. As especially patients with autism spectrum disorders (ASD) are assumed to exhibit a “weak central coherence”^53^, i.e. a detail-focused processing style, they might be predestined for future studies. That is, when considering this detail-focused processing style, it seems apparent that global Nogo-information should be less attended in ASD, thus suggesting a decreased response inhibition performance in this condition. Moreover, as patients with ASD are generally assumed to exhibit perceptual alterations in (multi-)sensory processing^54^, conducting this task in this specific subpopulation may grant the opportunity to further examine the effects of perceptual conflicts and corresponding ERPs. Furthermore, it might be interesting for future studies to additionally employ a fourth Nogo condition, by means of utilizing pop-out Nogo signals (i.e. to alter a single letter) on the local level in order to examine, whether this could result in local processing advantages and thus in improved response inhibition performance, especially in regard of above mentioned patients with ASD.

A limitation of this study is, however, that rather similar letter-stimuli were employed, which might thus have resulted in increased false alarm rates due to confusions, especially when considering the rather brief presentation times. However, as the behavioral data matched the electrophysiological data and moreover no differences in false alarm rates were evident in the global or local NoGo trials, which should differ in their susceptibility for confusions (see P1), this suggests that similar, but maybe attenuated, results would have been obtained, when utilizing less similar stimuli.

In summary, the study shows how perceptual conflicts modulate response inhibition processes. Information to inhibit a response is compromised by conflicting perceptual information, no matter whether the information to inhibit the response is carried via the global, or the local stimulus dimension. The neurophysiological data suggests that early perceptual/ attentional processing stages do however not underlie these modulations. Rather, processes at the response selection level, as displayed in the P3, play a role in the worsened response inhibition performance. Interestingly, this conflict-related impairment of inhibitory processes is associated with activation differences in (inferior) parietal areas (BA7 and BA40) and not as commonly found in the medial prefrontal areas. The results suggest that various functional neuroanatomical structures may mediate response inhibition processes and that the functional neuroanatomical structures involved are strongly modulated by the complexity of sensory integration processes involved.

## Materials and Methods

### Sample

N = 34 young healthy participants (20 females) between 20 and 29 years of age (mean age 23.4 ± 2.4 years) took part in the experiment. All participants had normal or corrected-to-normal vision and hearing, reported no neurological or psychiatric disorders and were free of any medication. Written informed consent was obtained from all participants. The study was conducted in accordance with the Declaration of Helsinki and was approved by the institutional review board of the Medical faculty of the TU Dresden.

### Task

**Figure 4:**
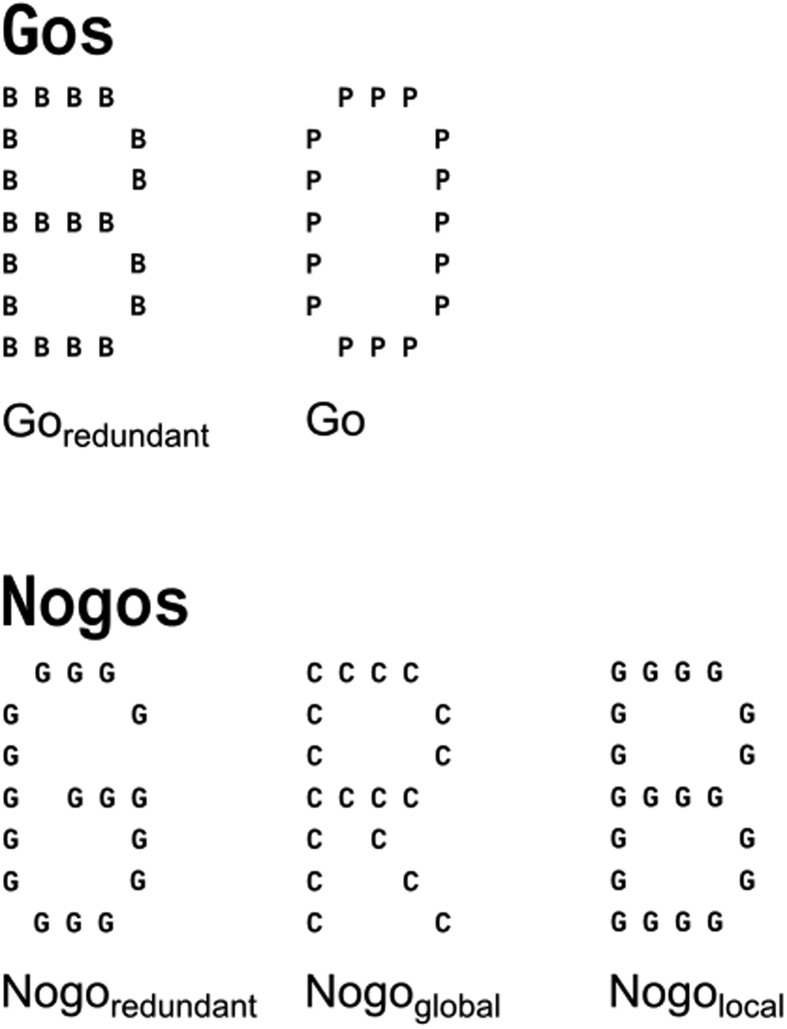
Stimulus material employed in the task. Upper row: examples for Go_redundant_ and Go stimuli. Lower row: Examples for Nogo_redundant_, Nogo_global_ and Nogo_local_ stimuli.

In this study, a newly developed combined Global-local-Go/Nogo task was applied. The task comprised 69% (412) Go trails and 31% (188) Nogo trials. As stimuli big (global) letters, composed of small (local level) letters, were utilized (refer Fig. 4).

Letters from the global and the local level could be non-conflicting or conflicting to each other. Letters within the local level were always identical. Two sets of letters were chosen, due to the visual similarity within each set (i.e. C, G and O (first set), and P, R and B (second set)). The two letters “G” and “R”, were used as Nogo stimuli, to ensure a maximal difficulty differentiating between Go and Nogo trials. When in the global (big letters), or the local (small letters) or on both levels the Nogo cues “G” or “R” were employed (“Gs” and “Rs” were never mixed), the trial was classified as a Nogo trial, in which participants had to refrain from responding. There are thus three different Nogo trials: If the letters at the global and local level were the same (i.e. non-conflicting), trials were coded as redundant Nogo trials (Nogo_redundant_). If Nogo cues were only present at the global level, Nogo trials were coded as global Nogo trials (Nogo_global_). If Nogo cues were only present at the local level, Nogo trials were coded as local Nogo trials (Nogo_local_). These latter two categories constituted the conflicting Nogo stimuli. If a response was executed on these Nogo trials within 1200 ms, this was treated as a false alarm.

On Go trials, that is on trials without Nogo cues in the presented letter(s), participants were instructed to press the space key with their dominant hand as fast as possible. There were two types of Go trials. If the letters at the global and local level were the same, trials were coded as redundant Go trials (Go_redundant_), if not, as Go trials (Go). Go trials were coded as hits, if a response was obtained within 1200 ms. Responses exceeding this deadline were regarded as misses. It is however not possible to examine the classical global precedence/interference effect^5^ on Go trials with this task, since the test’s conceptualization as a Go/NoGo task requires Go trials to be composed of Go cues on both the global and local level, which makes distinguishing local and global Go trials impossible.

Before the beginning of the experiment, a standardized exercise of 40 trials was conducted to familiarize participants with the stimuli. The experiment consisted of 600 trials with four blocks of 150 trials, each. Trial types (Go, Go_redundant_, Nogo_redundant_, Nogo_global_, Nogo_local_) were presented randomly, but it was ensured that all conditions were equally distributed across the blocks. Each trial began with the presentation of a letter (for 450 ms) centrally on the computer screen and subjects were required to press within 1200 ms (on Go trials). Each trial ended after 1700 ms. Trials were separated by inter-trial intervals (with fixation crosses) and jittered between 1100 and 1600 ms. The experiment lasted for about 25 minutes.

### EEG recording and analysis

EEG data was recorded from 60 Ag/AgCl electrodes arranged in equidistant positions. A sampling rate of 500 Hz was employed and electrode impedances were kept below 5 kΩ. Offline, a band-pass filter from 0.5 to 20 Hz (with a slope of 48 db/oct each) and a notch filter at 50 Hz were applied. Data was down-sampled to 256 Hz. Afterwards, an independent component analysis (ICA; infomax algorithm) was run for all participants on the un-epoched data sets in order to remove recurring artifacts. Only ICA components revealing horizontal and vertical eye movements, blinks and pulse artifacts were manually discarded. Afterwards, the EEG data was segmented (target-locked: −2000–2000 ms) for Go, Go_redundant_, Nogo_redundant_, Nogo_global_ and Nogo_local_ trials: Go trials were only taken into account when the correct response was given within 1200 ms after target onset. Likewise, Nogo trials were only included in subsequent data analysis when no response was given in the same time period. Subsequently, an automated artifact rejection procedure was conducted for all segments. A maximal value difference of 200 μV in a 100 ms interval and an activity below 0.5 μV in a 100 ms period and were used as rejection criteria. In order to eliminate reference potential from the data, a current source density (CSD) transformation^55^ was applied to re-reference the data. The resulting CSD values are stated in μV/m^2^. The CSD-transformation was employed, since it additionally serves as a spatial filter^55^. This makes it possible to identify electrodes that best reflect activity related to cognitive processes. Thereafter, a baseline correction from −200 ms to 0 ms prior to target onset was applied. For each category of segments, individual peaks were quantified semi-automatically for each participant. The resulting ERP components, namely P1 (at P7 & P8: 90–120 ms after target presentation onset), N1 (at P7 & P8: 155–185 ms) and N2 (at Cz: 270–400 ms) were identified by means of the scalp topography. For the P3 however, the mean activity in a time window of 450–600 ms was extracted at Pz based on the scalp topography. To validate this choice of electrodes for subsequent data analysis, the following procedure was applied^56^: The mean amplitudes of the ERP components in the corresponding search intervals (see above) at all electrode positions were extracted. Subsequently, each electrode was compared against an average of all other electrodes using Bonferroni-correction for multiple comparisons (critical threshold p = 0.0007). Based thereon, only electrodes showing significantly larger mean amplitudes than the remaining electrodes in at least one of the different experimental conditions (negative or positive) were chosen. Essentially, the electrodes chosen that way were coherent with those found in the visual data inspection. Peaks of all ERP components, except for the P3, were extracted to examine possible latency effects. Subsequent analyses on amplitudes were conducted based on the peak amplitudes. For the P3, the mean activity in the time window of 450–600 ms was used.

### Source localization analysis

Source localization was conducted using sLORETA (standardized low resolution brain electromagnetic tomography^57^). sLORETA reveals high convergence with fMRI and there also is evidence of from neuronavigated EEG/TMS studies underlining the validity of the sources estimated using sLORETA^58^,^59^. Moreover, it has been mathematically proven that this method provides reliable results without a localization bias^59^. sLORETA provides a single linear solution to the inverse problem, based on extra-cranial measurements without a localization bias^57^,^60^. For sLORETA, the intracerebral volume is partitioned into 6239 voxels at 5 mm spatial resolution. The standardized current density at each voxel is calculated in a realistic head model^61^ using the MNI152 template^62^. In this study, the voxel-based sLORETA images were compared across conditions using the sLORETA-built-in voxel-wise randomization tests with 2000 permutations, based on statistical nonparametric mapping (SnPM). Voxels with significant differences (p < 0.01, corrected for multiple comparisons) between contrasted conditions were located in the MNI-brain.

### Statistics

Behavioral data, i.e. Go reaction times (RTs) and frequency of Go hits was analyzed using dependent t-test. Frequency of Nogo false alarms (i.e., button presses on the different Nogo trials) were analyzed with a repeated-measures ANOVA using the within-subject factors “condition” (Nogo_redundant_, Nogo_global_, Nogo_local_). For the neurophysiological data, the factor “condition” (Go, Go_redundant_, Nogo_redundant_, Nogo_global_, Nogo_local_) was used as within-subject factor. When necessary, the factor “electrode site” was modeled as an additional within-subject factor. For all analyses Greenhouse-Geisser correction was applied wherever appropriate and all post-hoc tests were bonferroni-corrected. All variables analyzed were normal distributed (all z < 0.9; p > 0.3), as indicated by Kolmogorov-Smirnov tests. For the descriptive data, the mean and standard error of the mean (SEM) are given.

## Additional Information

**How to cite this article**: Chmielewski, W. X. and Beste, C. Perceptual conflict during sensorimotor integration processes - a neurophysiological study in response inhibition. *Sci. Rep.* **6**, 26289; doi: 10.1038/srep26289 (2016).
